# Identifiability and model selection frameworks for models of high-grade glioma response to chemoradiation

**DOI:** 10.1098/rsta.2024.0212

**Published:** 2025-04-02

**Authors:** Khushi C. Hiremath, Kenan Atakishi, Ernesto A. B. F. Lima, Maguy Farhat, Bikash Panthi, Holly Langshaw, Mihir D. Shanker, Wasif Talpur, Sara Thrower, Jodi Goldman, Caroline Chung, Thomas E. Yankeelov, David A. Hormuth II

**Affiliations:** ^1^ Department of Biomedical Engineering, The University of Texas at Austin, Austin, TX 78712, USA; ^2^ Texas Advanced Computing Center, The University of Texas at Austin, Austin, TX 78712, USA; ^3^ Oden Institute for Computational Engineering and Sciences, The University of Texas at Austin, Austin, TX 78712, USA; ^4^ Department of Radiation Oncology, MD Anderson Cancer Center, Houston, TX 77030, USA; ^5^ Faculty of Medicine, The University of Queensland, Brisbane, Queensland 4072, Australia; ^6^ Department of Imaging Physics, MD Anderson Cancer Center, Houston, TX 77030, USA; ^7^ Department of Diagnostic Medicine, The University of Texas at Austin, Austin, TX 78712, USA; ^8^ Department of Oncology, The University of Texas at Austin, Austin, TX 78712, USA; ^9^ Livestrong Cancer Institutes, The University of Texas at Austin, Austin, TX 78712, USA

**Keywords:** mathematical oncology, reaction–diffusion, model selection, image-based modelling

## Abstract

We have developed a family of biology-based mathematical models of high-grade glioma (HGG), capturing the key features of tumour growth and response to chemoradiation. We now seek to quantify the accuracy of parameter estimation and determine, when given a virtual patient cohort, which model was used to generate the tumours. In this way, we systematically test both the parameter and model identifiability. Virtual patients are generated from unique growth parameters whose growth dynamics are determined by the model family. We then assessed the ability to recover model parameters and select the model used to generate the tumour. We then evaluated the accuracy of predictions using the selected model at four weeks post-chemoradiation. We observed median parameter errors from 0.04% to 72.96%. Our model selection framework selected the model that was used to generate the data in 82% of the cases. Finally, we predicted the growth of the virtual tumours using the selected model resulting in low error at the voxel-level (concordance correlation coefficient (CCC) ranged from 0.66 to 0.99) and global level (percentage error in total tumour cellularity ranged from −12.35% to 0.07%). These results demonstrate the reliability of our framework to identify the most appropriate model under noisy conditions expected in the clinical setting.

This article is part of the theme issue 'Uncertainty quantification for healthcare and biological systems (Part 2)'.

## 1. Introduction

The field of mathematical oncology has matured to the point where it can now offer specific and personalized predictions of the dynamics of tumour growth and treatment response based on the biological characteristics of an individual patient’s tumour [1]. Once personalized, these modelling approaches can yield predictions of tumour outcomes for a variety of therapeutic interventions. Current models range in complexity from describing the changes in total tumour burden in response to treatment [2,3] to spatio-temporally resolved models of treatment response [4–6]. One particularly well-established area in predictive mathematical oncology, is in modelling high-grade glioma (HGG) growth and response to chemoradiation [7–9]. Despite an aggressive treatment strategy of surgery, radiotherapy (RT) and chemotherapy (CT), patients with HGG often have dismal prognoses and exhibit disease progression within 7−10 months [10]. The clinical need to improve patient outcomes has motivated many modelling studies aimed at characterizing tumour growth dynamics [11,12], predicting treatment response [6,13,14] and optimizing RT plans [15–17]. While these approaches have shown promising results in predicting and optimizing response [6,13–15], the identifiability of models and their parameters in the presence of noise is not well established for many of these models and computational frameworks for HGG. Especially in the clinical setting where there may be limited data, model and parameter identifiability is an important analysis to help determine if the proposed models (and their parameters) can be accurately determined given the available data. If the models and parameters are not identifiable given the data, approaches such as optimal experimental design and model reduction techniques [18–21] could be used to either adapt the data acquisition to the requirements of the model or the model to the available data. We note that there is a broad range of identifiability analyses [22–24]; however, here our parameter and model identifiability analysis systematically evaluate whether the appropriate model and its parameters can be determined from a given dataset.

With a disease as complex and heterogeneous as cancer, it is not always clear what the most appropriate model is to accurately describe (and predict) the desired quantity of interest [25]. To this end, we [6,26–30] and others [2,31–34] have built a model family where the members of the family capture the key characteristics of the investigated disease with varying assumptions and complexity. Statistical measures such as the Akaike information criteria (AIC) [35] can then be used to select the model that best balances model complexity (i.e. the number of parameters) and goodness of fit, and the selected model is then employed to predict tumour growth. In parallel to the model selection task, is the need to rigorously assess parameter identifiability [2,24,32] to determine if the models under consideration can be uniquely inverted for a given set of data. That is, a model (and its parameters) is deemed identifiable if a given set of parameter values result in a distinct distribution of the quantity of interest. A seminal work by Benzekry *et al*. [2], demonstrates this model selection, identifiability and prediction framework for eight classical models of tumour growth applied to two *in vivo* preclinical disease settings (Lewis lung carcinoma and breast carcinoma). A key finding of this study was that different mathematical models were selected for the two disease settings. In addition, the selected model (even if it was identifiable) did not necessarily deliver accurate predictions. Specifically, owing to varied growth stages (e.g. exponential growth to saturated growth), well-fitting models may under or overestimate future growth if the underlying model does not adequately describe the dynamics of the tumour during all stages of tumour growth. Similarly, Resende *et al*. [30] applied a model selection framework to 10 mathematical models to identify the appropriate model which described the *in vitro* dynamics of four different triple-negative breast cancer cell lines. The authors employed Bayesian information criterion to select the model which best described the data and observed that the selected model differed based on the cell line and treatment regimen. However, there was large uncertainty in selecting the most appropriate model for the data which the authors suggest could be reduced by combining datasets with identical treatment doses. More recently, Phillips *et al*. [27] applied a similar model selection, identifiability and prediction framework to four image-based mathematical models of breast cancer response to neoadjuvant therapy. Using a set of synthetic tumours, they demonstrated that in the presence of reasonable experimental noise, the correct model was identified for 70% of the cases and achieved less than 12.6% error in model parameter estimates. In the RT setting, Mohsin *et al*. [32] evaluated three different models of tumour response to RT in head and neck cancer and the implications of the selected model. As each model had varied assumptions on how RT results in tumour reduction, selection of the most appropriate model could have consequences on the optimization of patient-specific RT protocols. They observed that their quantity of interest (time to maximum reduction of tumour volume) varied between the three models and that parameter identifiability for a given parameter was dependent on the nominal values of the other parameters. Many of these challenges could be addressed through the use of a model calibration, selection and validation framework such as the Occam-plausibility algorithm (OPAL) [36]. OPAL employs an iterative approach to model calibration and selection and aims to identify the most parsimonious model determined to be valid. A model is considered valid if the predictions are within a pre-specified tolerance. By incorporating this validation step, OPAL ensures that a well-fitting model also delivers accurate predictions. Crucially, this step could identify models which may not accurately describe future tumour response. These five aforementioned studies highlight the insight model selection and parameter identifiability provided to predictive mathematical oncology studies. As inaccurate estimation of model parameters or selection of the incorrect model may have an effect on treatment decisions, these studies motivate the need for a similar investigation in the HGG setting.

In this contribution, we investigate the identifiability of a previously established family of models (and their parameters) describing the response of HGG to chemoradiation [6] in the setting of adaptive RT using an existing model calibration and selection framework from our preclinical [37,38] and clinical studies [6]. Adaptive RT [39–41] refers to a broad range of techniques that aim to adapt or change a patient’s RT plan to account for changes in the tumour geometry, location or underlying biology. In this study, we assume RT plans would be adapted to target changes in tumour geometry originating owing to tumour growth observed during RT. For such a scenario (e.g. clinical trial NCT# 04771806), patients are imaged for treatment planning, weekly during RT and at standard-of-care follow-ups. More specifically, we consider the modelling setting where a patient’s imaging data from treatment planning to week 3 (or mid-RT) are used to personalize model parameters to forecast tumour response at the first standard-of-care follow-up (i.e. four weeks post-RT). In this clinical setting, it is important to determine if the proposed models (and their parameters) are identifiable given the available data. As observed by others [2,32], multiple models may fit or describe the data used for calibration equally well but fail to predict or accurately characterize the relevant treatment-related parameters. While accurate predictions are important, as mathematical oncology moves towards the optimization of interventions [42], it is even more critical to identify the correct model (and its parameters) of response, as misidentifying the underlying biological mechanisms could adversely affect personalized treatment. For this study, we apply a similar approach as Phillips *et al*. [27] to a virtual cohort of patients whose growth and response parameters are sampled from physically relevant ranges, to determine the ability of our model framework to accurately recover model parameter values, as well as accurately identify the model used to generate the synthetic tumours for each patient within the virtual cohort. After performing model selection, we then also assess the accuracy of model predictions made with the selected models at the first, post-RT follow-up.

## 2. Methods

### (a) Family of mathematical models

Our model family consists of 11 members built upon the reaction–diffusion model of tumour growth [4,6,9] and considers 11 different assumptions on the effect of RT and/or CT on HGG. The reaction–diffusion model is a well-studied and established approach for medical-imaging-based models of tumour growth and response [4,6,9,43–46] and is used to model the highly invasive and diffuse infiltration of the tumour into the surrounding brain tissue (alternatively, for less diffuse diseases moving boundary approaches such as [47] could be used to model the interaction between the malignant and healthy appearing tumours. However here we assume diffuse infiltration into the healthy-appearing brain tissue). Throughout this section, the reader is encouraged to refer to table 1, where a summary of model parameters and variables is listed. Tumour growth and invasion are described using a coupled two-species reaction–diffusion model described by equations (2.1) and (2.2):


(2.1) $$\frac{\partial {\hat{N}}_{E}\left(x,t\right)}{\partial t}=\nabla \cdot \left(D_{E}\left(x,t\right)\nabla {\hat{N}}_{E}\left(x,t\right)\right)+k_{p,E}{\hat{N}}_{E}\left(x,t\right)\left(1-\left({\hat{N}}_{E}\left(x,t\right)+\beta_{NE}{\hat{N}}_{N}\left(x,t\right)\right)/\theta_{E}\right),$$

**Table 1.** Model variables and parameters.

parameter or variable	definition	source
*θ_j_*	carrying capacity for species *j*	*θ_j_* set to 1, *θ_N_* set to 0.16 [6,11]
*G_w_*	shear modulus for white matter	set to 2.7 kPa [48]
*G_g_*	shear modulus for grey matter	set to 3.1 kPa [48]
*ν*	Poisson’s ratio	set to 0.45
*λ_2_*	coupling constant	set to 1 N
$\vec{u}$	tissue displacement	calculated
*ER*	enhancement ratio	calculated
*σ_vm_*	von Mises stress	calculated
${\hat{N}}_{j}$	normalized tumour density for species *j*	measured from DWI
*n*	number of fractions of RT delivered	assigned from patient data
*Dose*	radiation therapy dose delivered during each visit	assigned from patient data
*k_p,E_*	proliferation rates for the enhancing tumour	calibrated
*k_p,N_*	proliferation rate for the non-enhancing tumour	calibrated
*D_j,w_,*	diffusion coefficient for white matter	calibrated
*D_j,g_*	diffusion coefficient for grey matter	calibrated
*f*_NE_	scaling factor for *D*_N_	calibrated
*β*_NE_	competition term of non-enhancing on enhancing	calibrated
*β*_EN_	competition term of enhancing or non-enhancing	calibrated
*λ*_1_	coupling constant	calibrated
*α*_RT_	RT efficacy term	calibrated
*α*_RT,prolif_	RT effect on proliferation term	calibrated
*α*_CT_	CT efficacy term	calibrated
*α/β*	ratio of linear and quadratic treatment terms	calibrated


(2.2) $$\frac{\partial {\hat{N}}_{N}\left(x,t\right)}{\partial t}=\nabla \cdot \left(D_{N}\left(x,t\right)\nabla {\hat{N}}_{N}\left(x,t\right)\right)+k_{p,N}{\hat{N}}_{N}\left(x,t\right)\left(1-\left({\hat{N}}_{N}\left(x,t\right)+\beta_{EN}{\hat{N}}_{E}\left(x,t\right)\right)/\theta_{N}\right),$$

where ${\hat{N}}_{i}$ is the normalized tumour density at a three-dimensional position ***x*** and time *t* for species *i* (= *E* or *N* indicating the enhancing or non-enhancing regions, respectively), *D_i_* is the tumour cell diffusion coefficient, *k_p,i_* is the tumour cell proliferation rate, *θ_i_* is the carrying capacity and *β_i,j_* is the competition parameter between species *i* and *j; D*_N_ was assigned as $D_{E}\cdot f_{NE}$ , where *f*_NE_ is a scaling factor relating the mobility of the enhancing component to the non-enhancing component of the disease. Enhancing and non-enhancing regions of the total tumour burden refer to the abnormal enhancement observed on post-contrast *T*_1_-weighted magnetic resonance imaging (MRI) and the abnormal hyperintense signal intensity observed on *T*_2_-weighted fluid-attenuated inversion recovery (*T*_2_-FLAIR) MRI, respectively. The enhancing tumour region is an area where the blood–brain barrier is disrupted and generally indicates areas of active tumour growth and neovascularization (contributing to the contrast enhancement) [49]. The non-enhancing region generally surrounds the enhancing tumour and reflects areas of tumour infiltration and peritumoral oedema. There are generally three main approaches used to describe tumour diffusion in image-based models: isotropic diffusion with varied coefficients assigned by tissue type [4,43]; coupling the spatially varying coefficients to mechanical tissue properties [6,50,51]; and non-isotropic diffusion with diffusion tensor at each location within the domain [52,53]. Here, we spatially and temporally evolve *D_i_* as a function of local tissue mechanical properties, via


(2.3) $$D_{i}\left(x,t\right)=D_{i,0}\left(x\right)exp\left(-\lambda_{1}\sigma_{vm}\left(x,t\right)\right),$$

where *D_i,0_* is the unrestricted diffusion coefficient assigned unique values for white and grey matter, *λ_1_* is the stress-tumour cell diffusion coupling constant and *σ*_vm_ is the von Mises stress (a measure of the total, local stress). As *σ*_vm_ increases and approaches infinity, tumour cell mobility is inhibited, reducing *D_i_* to zero. Conversely, as *σ*_vm_ decreases towards zero, tumour cell mobility is unrestricted, resulting in *D_i_* = *Di_i,0_*. The von Mises stress is determined by solving the linear elastic mechanical equilibrium equation for tissue displacement $\vec{u}$ :


(2.4) $$\nabla \cdot G\nabla \vec{u}+\nabla \frac{G}{1-2\upsilon }\left(\nabla \cdot \vec{u}\right)-\lambda_{2}\nabla \left({\hat{N}}_{N}\left(x,t\right)+{\hat{N}}_{E}\left(x,t\right)\right)=0,$$

where *ν* and *G* are literature values of Poisson’s ratio and shear modulus, respectively, and *λ_2_* is the second scaling constant set to 1 N m. The calculated tissue displacement is then used to calculate the stress tensor using Hooke’s law (included in the electronic supplemental material). Complete details can be found in [37,50,54].

RT and CT were modelled as a discrete reduction in ${\hat{N}}_{i}$ using


(2.5) $${\hat{N}}_{i,post}\left(x,t\right)={\hat{N}}_{i,pre}\left(x,t\right)SF_{RT}\left(x,t\right)SF_{CT}\left(x,t\right),$$

where ${\hat{N}}_{i,post}$ is the normalized tumour density immediately following the treatment event, ${\hat{N}}_{i,pre}$ is the normalized tumour density immediately preceding the treatment event and SF_RT_ and SF_CT_ are the surviving fractions of the tumour owing to RT and CT, respectively. We also considered a prolonged effect of RT exposure which results in a reduced fraction of actively proliferating tumour cells [38,55] described via


(2.6) $$k_{p,i}=k_{p,0}SF_{RT,prolif}{\left(x,t\right)}^{n},$$

where *k_p,_*_0_ is the patient-specific proliferation rate, SF_RT,prolif_ is the reduction of the proliferation rate per single fraction of RT, and *n* is the number of fractions delivered.

Using the base model described in equations (2.1)–(2.6), we considered 11 different assumptions of the efficacy of RT and CT on tumour cells investigated by us [6,38,56] and others [13,57]. These 11 different assumptions constitute our 11 different models labelled *M*_1_ to *M*_11_. Models *M*_1_ and *M*_2_ consider spatially uniform efficacy of both RT and CT (*M*_1_) or on a combined RT/CT term (*M*_2_). The combined RT/CT term ignores the effect of a separate CT term by setting SF_CT_ to 1. Models *M_3_–M_6_* vary the efficacy of RT (*M_3_*), CT (*M*_4_), RT and CT (*M_5_*) and RT/CT (*M_6_*) as a function of tumour vasculature (as estimated via the enhancement ratio). Models *M*_7_ *–M*_10_ vary the efficacy of RT (*M*_7_), CT (*M*_8_), RT and CT (*M*_9_) and RT/CT (*M*_10_) as a function of cell density. In models *M*_3_, *M*_4_, *M*_7_ and *M*_8_, we vary the effect of vasculature or cell density on each treatment modality to assess whether we distinguish differences in treatment effects. This approach also allows for scenarios where the coupling term of interest may better reflect CT delivery than RT sensitivity, and vice versa. Finally, *M*_11_ ignores the effect of treatment altogether and sets SF_RT_, SF_CT_ and SF_RT,prolif_ to 1; SF*_i_* for models *M*_1_ *–M*_10_ are derived from the linear quadratic model of response to RT shown in equation (2.7):


(2.7) $${\text{SF}}_{i}\left(x,t\right)=e^{-\alpha_{i}\cdot \text{Dose}\left(x,t\right)\left(1+\frac{\text{Dose}\left(x,t\right)}{\alpha /\beta }\right)},$$

where *α_i_* is a treatment sensitivity term for treatment modality *i*, Dose(***x*** *,t*) is the dose of RT given in a single fraction and *α*/*β* is the ratio of the linear and quadratic sensitivity terms. Note that, for the CT term, *α*/*β is* set to zero, while for the RT term, the *α*/*β* ratio is calibrated. Models *M*_1_ and *M*_2_ assign treatment efficacy as equation (2.7). In models *M*_3_ *–M*_6_ where we relate treatment efficacy to tissue vascularization, we modify equation (2.7) to account for tissue vascularization which we assume is proportional to tissue oxygenation, using equations (2.8) and (2.9):


(2.8) $${\text{SF}}_{i}\left(x,t\right)=e^{-\frac{\alpha \cdot \text{Dose}\left(x,t\right)}{\text{OER}\left(x,t\right)}\left(1+\frac{\text{Dose}\left(x,t\right)}{\text{OER}\left(x,t\right)\cdot \alpha /\beta }\right)},$$

(2.9) OER(x,t)=max(ER(x,t))−ER(x,t)+1,

where OER(***x*** *,t*) is the oxygen enhancement ratio and ER(***x*** *,t*) is the ratio of the post-contrast *T*_1_-weighted to the pre-contrast *T*_1_-weighted MRI. A voxel that is well-perfused (and we assume well-oxygenated) results in an OER of 1, whereas a poorly perfused voxel has an OER that exceeds 1 (decreasing the efficacy of the treatment). For models *M*_7_ *–M*_10_, we modulate the surviving fraction calculated in equation (2.7) as a function of the normalized tumour density using equation (2.10):


(2.10) $${SF}_{i}\left(x,t\right)={SF}_{i,min}+\left(1-\text{S}{\text{F}}_{i,min}\right)\left(1-\frac{\sum_{1}^{2}{\hat{N}}_{j}\left(x,t\right)}{\sum_{1}^{2}\theta_{j}}\right),$$

where $SF_{i,min}$ is the minimum surviving fraction calculated using equation (2.7), ${\hat{N}}_{j}$ is the normalized tumour density for species *j*, and *θ_j_* is the carrying capacity for species *j*. Equation (2.10) assumes that as a voxel reaches its carrying capacity (i.e. the maximum number of cells a tissue can physically and biologically support), it begins to proliferate less frequently and therefore is less susceptible to death via mitotic catastrophe. Table 2 lists the model family, assumptions on treatment efficacy, and the number of calibrated parameters.

**Table 2.** Summary of model calibration and prediction accuracies.

			calibration (mid-RT)			prediction (4 weeks post-RT)		
	**model**	**noise**	CCC	DSC	PE_TTC_	CCC	DSC	PE_TTC_
uniform efficacy	***M*_1_** RT & CT 12	5%	0.97 (0.09)	0.99 (0.01)	−0.00 (0.06)	0.91 (0.22)	0.98 (0.13)	−1.73 (4.73)
		10%	0.89 (0.27)	0.99 (0.03)	−0.01 (0.20)	0.84 (0.10)	0.97 (0.20)	−2.12 (8.91)
		15%	0.77 (0.41)	0.98 (0.04)	−0.02 (0.28)	0.71 (0.26)	0.94 (0.25)	−2.55 (10.98)
	***M*_2_** RT/CT 11	5%	0.96 (0.08)	1.00 (0.01)	−0.01 (0.08)	0.76 (0.41)	0.98 (0.05)	−4.00 (14.20)
		10%	0.86 (0.24)	0.99 (0.01)	−0.03 (0.45)	0.70 (0.32)	0.98 (0.10)	−6.62 (14.79)
		15%	0.76 (0.33)	0.98 (0.02)	−0.05 (0.87)	0.62 (0.13)	0.98 (0.15)	−9.07 (15.88)
coupled to vasculature	***M*_3_** RT 12	5%	0.98 (0.02)	0.99 (0.03)	0.00 (0.05)	0.95 (0.19)	0.98 (0.05)	−7.71 (22.51)
		10%	0.94 (0.06)	0.98 (0.07)	−0.01 (0.09)	0.88 (0.20)	0.96 (0.07)	−8.40 (19.73)
		15%	0.86 (0.11)	0.96 (0.10)	−0.03 (0.12)	0.77 (0.14)	0.95 (0.08)	−8.26 (19.73)
	***M*_4_** CT 11	5%	0.97 (0.06)	0.99 (0.02)	−0.02 (0.13)	0.91 (0.19)	0.99 (0.08)	−1.79 (9.01)
		10%	0.84 (0.23)	0.98 (0.04)	−0.04 (0.22)	0.70 (0.36)	0.98 (0.14)	−4.88 (8.90)
		15%	0.70 (0.33)	0.97 (0.07)	−0.07 (0.27)	0.79 (0.45)	0.98 (0.08)	−3.01 (11.48)
	***M*_5_** RT & CT 12	5%	0.98 (0.01)	1.00 (0.01)	−0.02 (0.10)	0.75 (0.58)	0.96 (0.33)	−10.05 (23.44)
		10%	0.94 (0.03)	0.99 (0.01)	−0.04 (0.25)	0.77 (0.57)	0.96 (0.14)	−6.91 (10.59)
		15%	0.88 (0.07)	0.99 (0.02)	−0.03 (0.35)	0.65 (0.49)	0.94 (0.25)	−11.22 (24.08)
	***M*_6_** RT/CT 12	5%	0.99 (0.01)	1.00 (0.00)	0.01 (0.05)	0.98 (0.05)	0.99 (0.04)	−2.08 (5.02)
		10%	0.95 (0.04)	1.00 (0.01)	0.01 (0.08)	0.94 (0.10)	0.98 (0.05)	−2.83 (6.64)
		15%	0.89 (0.08)	0.99 (0.02)	−0.01 (0.14)	0.89 (0.11)	0.97 (0.12)	−2.94 (6.50)
coupled to cell density	***M*_7_** RT 12	5%	0.97 (0.03)	1.00 (0.01)	−0.00 (0.04)	0.96 (0.25)	1.00 (0.00)	−0.13 (4.73)
		10%	0.90 (0.10)	0.99 (0.01)	−0.01 (0.11)	0.83 (0.38)	1.00 (0.01)	−0.39 (7.56)
		15%	0.80 (0.18)	0.99 (0.06)	−0.01 (0.24)	0.71 (0.45)	0.99 (0.02)	−0.24 (5.10)
	***M*_8_** CT 11	5%	0.97 (0.02)	1.00 (0.01)	−0.01 (0.06)	0.93 (0.43)	0.97 (0.39)	−2.26 (12.89)
		10%	0.89 (0.07)	0.99 (0.03)	−0.02 (0.10)	0.75 (0.56)	0.92 (0.59)	−6.30 (19.60)
		15%	0.77 (0.12)	0.98 (0.04)	−0.07 (0.18)	0.60 (0.62)	0.87 (0.60)	−10.11 (28.69)
	***M*_9_** RT & CT 12	5%	0.98 (0.02)	1.00 (0.01)	0.00 (0.04)	0.95 (0.29)	1.00 (0.01)	−1.52 (6.18)
		10%	0.93 (0.06)	1.00 (0.01)	−0.00 (0.10)	0.89 (0.44)	1.00 (0.01)	−3.60 (10.27)
		15%	0.85 (0.12)	0.99 (0.02)	−0.00 (0.14)	0.78 (0.47)	1.00 (0.01)	−3.62 (10.71)
	***M*_10_** RT/CT 12	5%	0.98 (0.02)	1.00 (0.00)	0.02 (0.04)	0.97 (0.06)	1.00 (0.00)	−0.05 (2.14)
		10%	0.93 (0.07)	0.99 (0.01)	0.02 (0.10)	0.89 (0.12)	1.00 (0.00)	−0.06 (2.16)
		15%	0.85 (0.12)	0.99 (0.01)	0.04 (0.13)	0.78 (0.14)	1.00 (0.01)	−0.10 (2.23)
no treatment	***M*_11_** — 8	5%	0.99 (0.02)	1.00 (0.00)	0.02 (0.05)	0.97 (0.05)	1.00 (0.00)	−0.02 (0.49)
		10%	0.96 (0.07)	1.00 (0.00)	0.05 (0.09)	0.91 (0.13)	1.00 (0.00)	−0.04 (0.39)
		15%	0.91 (0.14)	1.00 (0.00)	0.05 (0.12)	0.83 (0.22)	1.00 (0.00)	−0.06 (0.49)

Equations (2.1)–(2.10) were numerically solved using a three-dimensional finite-difference approximation implemented in MATLAB R2022a on a computational domain discretized with a resolution of 0.5 × 0.5 × 2 mm (appropriate for high-resolution *in vivo* MRI data). Zero-flux boundary conditions were assumed for normalized tumour density at the skull boundary (i.e. $\nabla {\hat{N}}_{E}\cdot n=0$ $\nabla {\hat{N}}_{N}\cdot n=0$ ), while $\vec{u}$ boundary conditions allowed tangential displacement (slip condition) but not normal displacement ( $\vec{u}\cdot n$ = 0).

### (b) Generation of virtual patient cohort

For each model, *M_1_* to *M_11_*, a synthetic tumour was generated using unique initial conditions of ${\hat{N}}_{E}\left(x,t\right)$ and ${\hat{N}}_{N}\left(x,t\right)$ and anatomy from 21 patients with histologically confirmed Glioblastoma, negative for IDH 1 and IDH 2 mutation (i.e. IDH wild-type), who had undergone maximal safe surgical resection followed by concurrent adaptive RT and Temozolomide as per the Stupp protocol [58]. The RT prescription is 60 Gy to the gross tumour volume and then 50 Gy to the gross tumour volume with a 2 cm margin for the clinical target volume [59] over a six-week period. Typically, RT is delivered in 30 fractions, administered daily from Monday to Friday over a period of six weeks. The 21 patients were enrolled in NCT# 04771806, a single-arm prospective clinical trial approved by the institutional review board at The University of Texas M.D. Anderson Cancer Center (IRB# PA17-0844). All components of the clinical trial were performed in accordance with relevant guidelines and regulations and informed consent was collected from eligible patients who were enrolled into this trial.

In this study, only the multi-parametric MRI data collected for RT treatment planning were used to generate initial conditions for 21 unique virtual patients. Specifically, pre- and post-contrast *T*_1_-weighted MRI was used to label the abnormal enhancement as enhancing tumour, while *T*_2_-weighted fluid-attenuated inversion recovery (*T*_2_-FLAIR) was used to label abnormal hyperintense signal intensity as a non-enhancing tumour. Segmenting these regions was performed within Raystation 10 B DTK or 11 B DTK (RaySearch Laboratories, Stockholm, Sweden) by a trained expert and approved by a senior radiation oncologist. Using previously established approaches [5,6], ${\hat{N}}_{E}\left(x,t\right)$ was calculated within the enhancing tumour region using diffusion-weighted MRI estimates of the apparent diffusion coefficient, ADC, via


(2.11) $${\hat{N}}_{E}\left(x,t\right)=\frac{ADC_{w}-ADC\left(x,t\right)}{ADC_{w}-ADC_{min}},$$

where ADC_w_ is the ADC of water (set to 3 × 10^–3^ mm^2^ s^−1^; [60], and ADC_min_ is the minimum ADC observed within the tumour across all patients and time points; ${\hat{N}}_{N}\left(x,t\right)⁠$ was set to a fixed value of 0.16 [6,11] within the non-enhancing disease. Fractional anisotropy and ADC maps were then used to segment white and grey matter using a two-step segmentation approach [61] and *k-*means clustering. First, the ADC maps are segmented into ‘high ADC’ (consisting of cerebral spinal fluid, necrotic regions or resection cavities) and ‘low ADC’ (including healthy-appearing brain tissue and tumour tissues). Next, within the low ADC region, we apply a second *k*-means clustering on the fractional anisotropy maps to segment white matter and grey matter. The segmented white and grey matter maps are used to assign spatially varying mechanical properties and diffusion coefficients. *T*_1_-weighted MRI was used to identify the brain-skull interface using skull stripping [62]. The segmented white matter, grey matter and the entire brain were used to define the computational domain for each patient.

For each patient-specific initial condition (e.g. ${\hat{N}}_{E}\left(x,t=0\right)$ and ${\hat{N}}_{N}\left(x,t=0\right)$ ) and computational domain, we generated a synthetic tumour for each model (*M*_1_ *–M*_11_) with a unique set of model parameters via equations (2.1)–(2.10). The model parameters were sampled from within the ranges reported in the electronic supplementary material, table S1. This yielded a total of 231 synthetic tumours for which the growth and response to chemoradiation were described by a unique set of model parameters (i.e. 231 different sets of model parameters). We opted for a mixture of patient-specific experimental data (i.e. initial conditions, anatomy, vasculature/perfusion maps and treatment regimens) and synthetic data (i.e. tumour-specific model parameters) to create a heterogenous cohort of virtual patients with realistic distributions of tumour cells. To reduce the computational cost, memory and storage requirements necessary to perform the large number of numerical experiments described in §2c, the computational domain consisted of the three slices centred around the largest tumour slice observed in the initial condition. As the emphasis of this paper is on assessing the uncertainty in parameter estimates and predictions for synthetic tumours rather than the patient’s actual data, the three-slice approach bridges the two-dimensional approach used previously [27] and a larger three-dimensional approach. As the computational domain is set prior to ‘growing’ the synthetic tumour, no tumour growth data is lost by reducing computational domain. In addition to the initial condition time point the synthetic tumour was sampled (or ‘imaged’) at the beginning of each of the first three weeks of RT, and at four weeks post-RT. This yielded five three-dimensional distributions of tumour burden: one pre-treatment, three during treatment and one post-treatment. All computations were performed on the Lonestar 6 high-performance computing cluster at the Texas Advanced Computing Center where each compute node consisted of dual AMD EPYC 7763 CPUs with shared 256 GB memory.

### (c) Parameter and model identifiability analyses

**Figure 1.**
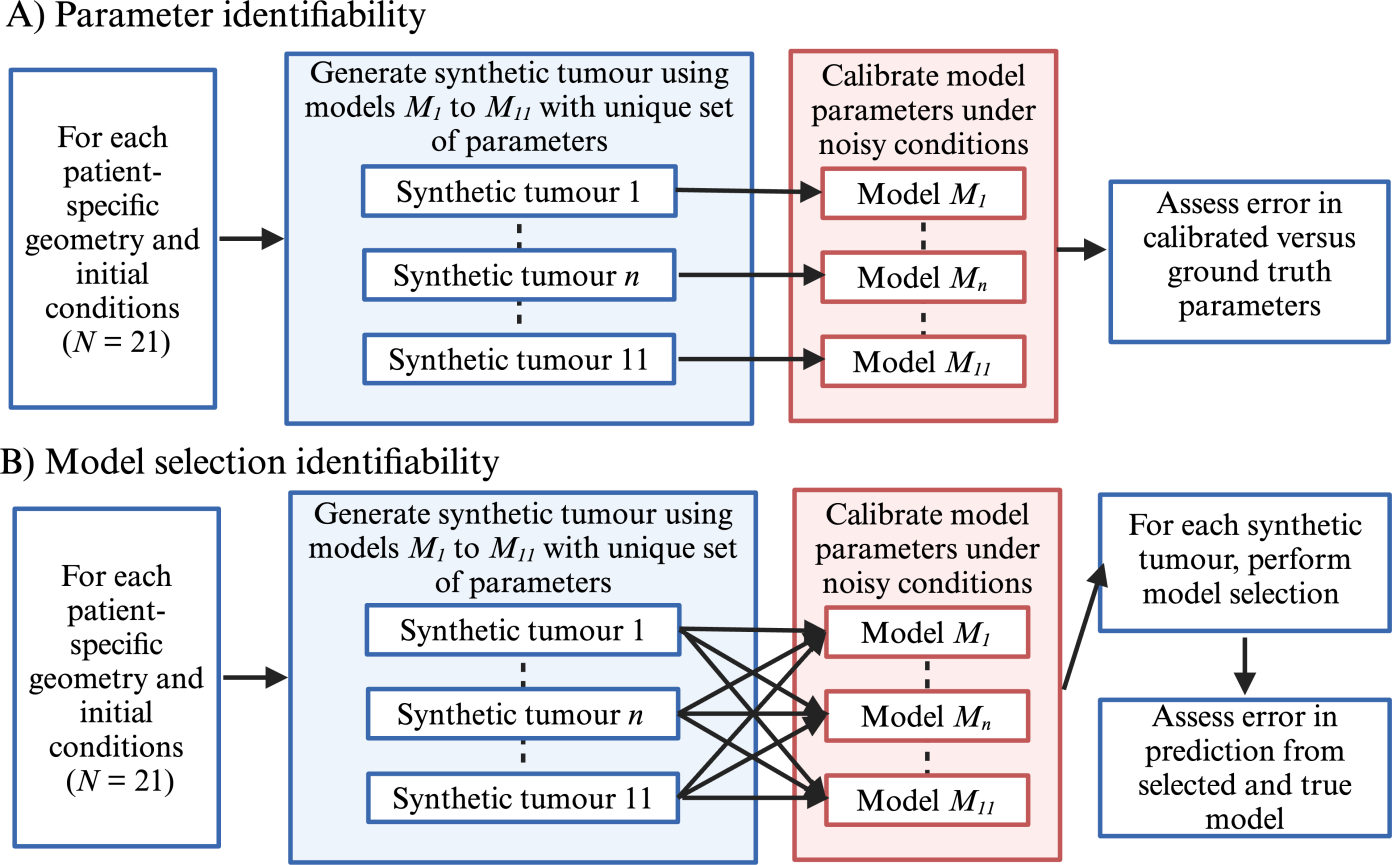
Schematic of the parameter and model identifiability analyses. Panel (A) shows the workflow of analysing the error associated with estimating model parameters under noisy conditions (5%, 10% and 15% added noise). For each patient-specific geometry and initial condition, synthetic tumours were generated with a unique set of model parameters for each model. We then calibrated each model to the corresponding synthetic tumour under noisy conditions. The error between calibrated and ground truth parameters was assessed. Panel (A) shows the workflow for assessing the identifiability of the model selection framework. The workflow for model selection identifiability follows the same path as Panel (A) until the calibration stage. Here, we calibrated all 11 models to each synthetic tumour and performed model selection to identify the model that best represented the data. Using the selected model, we then assessed the error in the predicted tumour growth at follow-up visits. Created with BioRender.com.

Figure 1 shows a schematic of the overall parameter and model identifiability analyses [27,63]. We first analysed parameter identifiability (figure 1A) by calibrating each synthetic tumour (*n* = 231) with the model used to generate the tumour under three noise levels (5%, 10% and 15% Gaussian noise added to the data) and calculated the percentage error between the calibrated and known, or ground truth parameters. Fifteen per cent of Gaussian noise represents the upper end of expected noise in clinical *in vivo* MRI studies [27]. For each synthetic tumour, up to 12 parameters (see table 2 for the number of parameters calibrated per model) were calibrated using the Levenberg–Marquardt algorithm applied to data from pre-treatment to week 3 of RT. For models *M*_2_, *M*_4_ and *M*_8_ *α*_CT_ is set to 1 and is not calibrated. For model *M*_11_, *α*_CT_, *α*_RT_, *α*_RT,prolif_ and *α/β* are not used. The remaining models use all the parameters listed as ‘calibrated’ in table 1. The Levenberg–Marquardt algorithm is a deterministic, gradient-based approach that switches between the Gauss–Newton and gradient descent algorithms to minimize the sum of squared errors. We define our nonlinear least-squares approach as follows:


(2.12) $$Y\left(x_{i},t\right)=f\left(x_{i},t,\beta \right)+\varepsilon ,$$

where Y are our observations at position *x*_i_ and time *t*, $f(x_{i},t,\beta )$ is our model evaluated with parameters *β* and temporally independent normal noise model (i.e. $\varepsilon \;∼\;N\left(0,\sigma^{2}\right)$ ; ε is the error term, $N\left(0,\sigma^{2}\right)$ denotes a Normal distribution with a variance of $\sigma^{2}$ ). The Levenberg–Marquardt algorithm seeks to find the set of parameters *β* that minimizes the residual sum of squared errors (RSS):


(2.13) $$\text{RSS}\left(\beta \right)=\sum_{t=2}^{4}\sum_{i=1}^{n}{\left(Y\left(x_{i},t\right)-f\left(x_{i},t,\beta \right)\right)}^{2}.$$

Under the assumptions of constant variance and temporally independent noise, this formulation is equivalent to maximizing the maximum likelihood (*L*) estimation via a Bayesian approach with uniform priors:


(2.14) $$\log L\left(\beta ,\sigma^{2}\right)=-\frac{n}{2}\log2\pi \sigma^{2}-\frac{1}{2\sigma^{2}}\sum_{t=2}^{4}\sum_{i=1}^{n}{\left(Y\left(x_{i},t\right)-f\left(x_{i},t,\beta \right)\right)}^{2}.$$

A detailed implementation of the Levenberg–Marquardt algorithm may be found in [5,54], but briefly: in our implementation, the algorithm [5,54,64] iteratively updated model parameters until the sum of the squared errors between the ground truth synthetic tumour and model-estimated tumour during the first three weeks of RT was minimized. The algorithm continued until either the maximum number of iterations, 250, was reached or when the change in parameter values between successive iterations was below 0.01%. Each calibration was initialized with the measured tumour cell density and an initial guess of model parameters from the middle of the parameter bounds (electronic supplementary material, table S1). For our model identifiability analyses (figure 1B), we calibrated each synthetic tumour with all 11 models under three noise levels (a total of 7623 calibrations), performed model selection and then assessed the error in the predicted tumour response at the four weeks post-RT time point. The AIC was used to select the most parsimonious model that balanced model complexity and agreement with the observations:


(2.15) $$\text{AIC}=2k+n\ln\left(\frac{\text{RSS}}{n}\right)+2k\left(\frac{k+1}{n-k-1}\right),$$

where *k* is the number of calibrated parameters for a given model, *n* is the number of data points used for calibration and RSS is the residual sum of squared errors between the ground truth synthetic tumour and model-estimated tumour growth. The AIC is a widely used method that can be applied to both nested and non-nested models. As some of the models in this study are not nested, we opted for the AIC over using paired likelihood ratio tests, which are typically applied to nested models. The model with the minimum AIC was then selected as the optimal model. This process was repeated for all synthetic tumours and at all noise levels. To quantify the ability of our formalism to accurately select the model that was used to construct the data, we tabulated the number of times each model was correctly and incorrectly selected as the model that generated the tumour.

We note that Bayesian inference methods are frequently applied for identifiability studies (as in [24,27]) in place of the least-squares-based approach used in this study. Our motivation for using a Levenberg–Marquardt approach for solving the least-squares problem is rooted in the ability of our existing calibration and selection framework [5,6] to accurately recover model parameters and select the model that ‘grew’ the synthetic tumour. Our current framework uses the Levenberg–Marquardt method for estimating parameters and the AIC for model selection. This is a practical computational choice as it is able to return model calibrations and predictions in a clinically relevant time frame (i.e. less than 24 h) for all models in our complete model family (some of which have hundreds to thousands of parameters) and on much larger computational domains. This is potentially a limitation as our current framework (compared to a Bayesian framework) does not directly account for the uncertainty in the underlying data and model that is undoubtedly important for establishing confidence and trustworthiness of model predictions. This point is revisited in the §4.

### (d) Statistical analysis

For the first analysis (figure 1A), the percentage error between the calibrated and ground truth parameters used to generate the synthetic tumour was calculated and reported for each model and noise level. For the second analysis (figure 1B), the accuracy of the model calibrations and predictions were assessed at the global and voxel levels at both week 3 of RT (i.e. the last time point used for calibration) and at four weeks post-RT (i.e. the prediction time point). At the global level, the Dice similarity coefficient (DSC) was calculated to measure the degree of spatial overlap between the synthetic and model-estimated tumours. The DSC ranges from zero (no overlap) to 1 (perfect overlap) and is calculated using equation (2.16):


(2.16) $$\text{DSC}=2\frac{\left|V_{synthetic}\cap V_{model-estimated}\right|}{\left|V_{synthetic}\right|+\left|V_{model-estimated}\right|},$$

where *V*_synthetic_ and *V*_model-estimated_ are two binary masks of the synthetic and model-estimated volumes. In addition, the percentage error between the synthetic and model-estimated total tumour cellularity (TTC) was calculated using equation (2.17):


(2.17) $$TTC=\theta_{cells}\sum_{x=1}^{n}\left({\hat{N}}_{N}\left(x,t\right)+{\hat{N}}_{E}\left(x,t\right)\right),$$

where *θ*_cells_ is the physical carrying capacity in terms of the number of cells that can fit in a given voxel and *n* is the number of voxels within the computational domain; *θ*_cells_ is calculated [5] assuming a packing density of 0.7405 [65], an verage cell radius of 10 μm and a voxel volume of 0.5 mm^3^. At the voxel level, the level of spatial agreement between the synthetic and model-estimated combined normalized tumour cell density (i.e. ${\hat{N}}_{E}\left(x,t\right)+{\hat{N}}_{N}\left(x,t\right)$ ) was assessed using the concordance correlation coefficient (CCC) [66] calculated using equation (2.18):


(2.18) $$\text{CCC}=\frac{2s_{ab}}{s_{a}^{2}+s_{b}^{2}+{\left(\overset{-}{a}+\overset{-}{b}\right)}^{2}}\;,$$

where *a* and *b* represent the synthetic and model-estimated combined normalized tumour cell density, *s_ab_* is the covariance between variables, $s_{i}^{2}$ is the variance for variable *i* and $\overset{-}{i}$ is the mean for variable *i*. The CCC ranges from −1 (perfect disagreement) to 1 (perfect agreement). We used the median and interquartile range (IQR) to summarize the error metrics central tendency and variability, respectively. Median and IQR were used as not all error metric distributions followed a normal distribution, as determined using the Lilliefors test in MATLAB.

## 3. Results

### (a) Parameter identifiability

**Figure 2.**
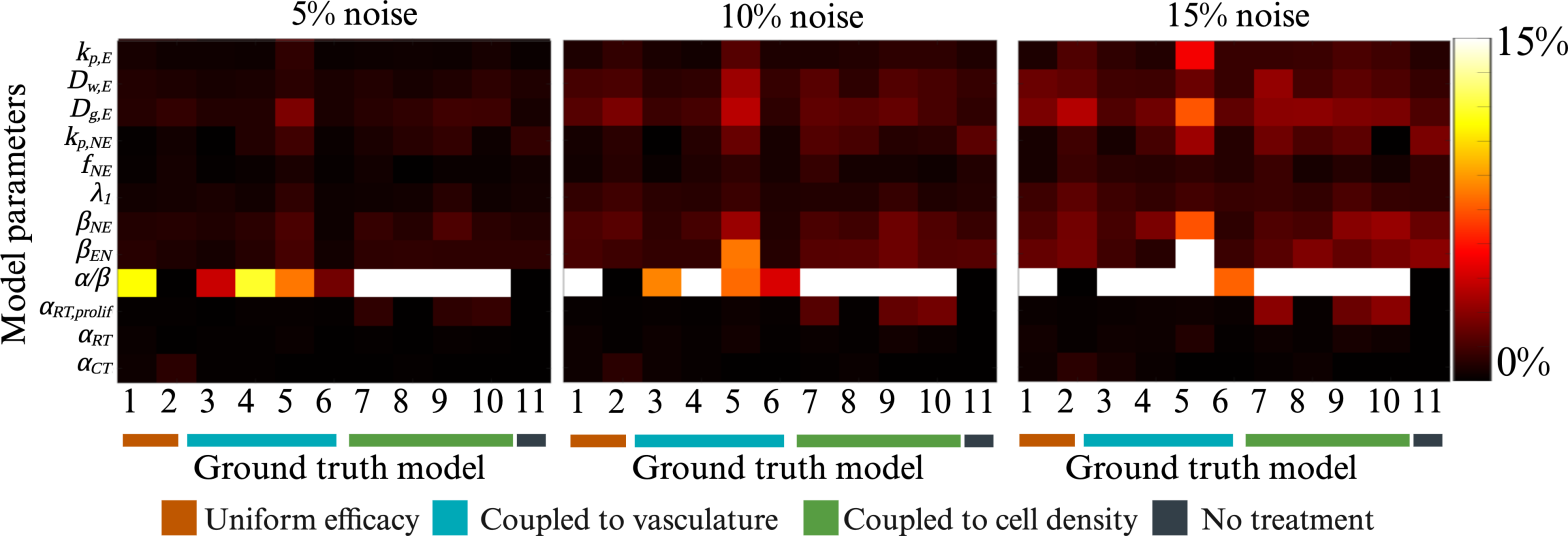
Identifiability of model parameters from noisy data. Median percentage errors in model parameters (vertical axis) are reported for each model (horizontal axis) estimated under three conditions of experimentally relevant noise (5%, 10% and 15%). We note that the maximum value for each colour map is set 15% to facilitate visualization of lower levels of error in the estimated parameters. For most models and all three noise levels, the *α*/*β* ratio had the poorest identifiability with the median error ranging from 2.47 to 72.96%. The remaining parameters had between 0 and 2.73% error for 5% noise, 0.07% and 8.21% error for 10% noise and 0.11% and 18.59% error for 15% noise. Created with BioRender.com.

Figure 2 shows the results of the parameter identifiability analysis for all three noise levels. Generally, the median percentage error in estimated model parameters increased with increasing noise. More specifically, the median error across all parameters was 0.49% (IQR = 0.76%), 0.95% (IQR = 1.42%) and 1.30% (IQR = 1.91%) for the three noise levels, respectively. The overall median of all the parameters was 0.85% (IQR = 1.36%, values ranged from 0 to 72.96%). Notably, the treatment terms *α*_RT_ and *α*_CT_ both had the lowest error across all three noise levels with the median percentage error in noise ranging from 0.11 to 0.40%. The *α*/*β* ratio, however, had the greatest median percentage error of all parameters with errors ranging from 2.47 to 72.96% for all noise levels. Model *M*_6_ had the lowest median error across all the parameters with a median error ranging from 0.24% (IQR = 0.34%) to 1.19% (IQR = 0.49%) for all noise levels. Similarly, model *M*_5_ had the highest median error across all the remaining parameters with a median error ranging from 1.01% (IQR = 0.94%) to 3.41% (IQR = 6.35%) for all noise levels (electronic supplementary material, tables S2 and S3, report the median and IQR for all results shown in figure 2).

### (b) Model selection identifiability

**Figure 3.**
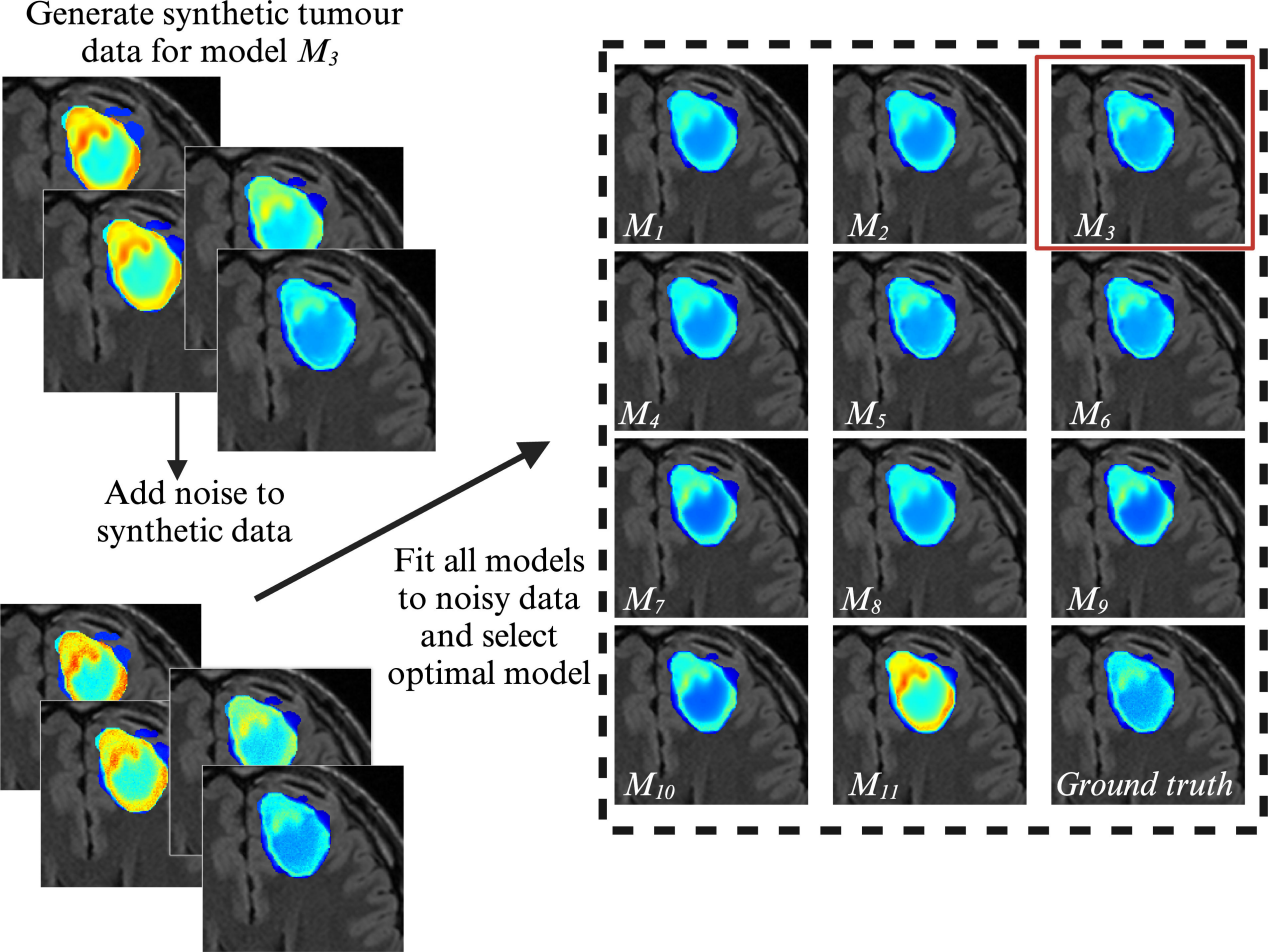
Visualization of model selection framework for a given model *M*_3_. For a given patient-specific geometry and initial condition, a synthetic tumour time course is generated for model *M*_3_. Noise ranging from 5% to 15% is then added to the synthetic data. Each model is then calibrated to the noisy data, after which the AIC is used to select the optimal model. The images within the black-dashed rectangle show the central slice of the tumour cell distribution mid-RT for all 11 members of the model family and the ground truth used to calibrate the models. The model in the red box shows the model that was selected using the AIC. Model *M*_3_ was also used to generate the ‘ground truth’ in this example. Created with BioRender.com.

**Figure 4.**
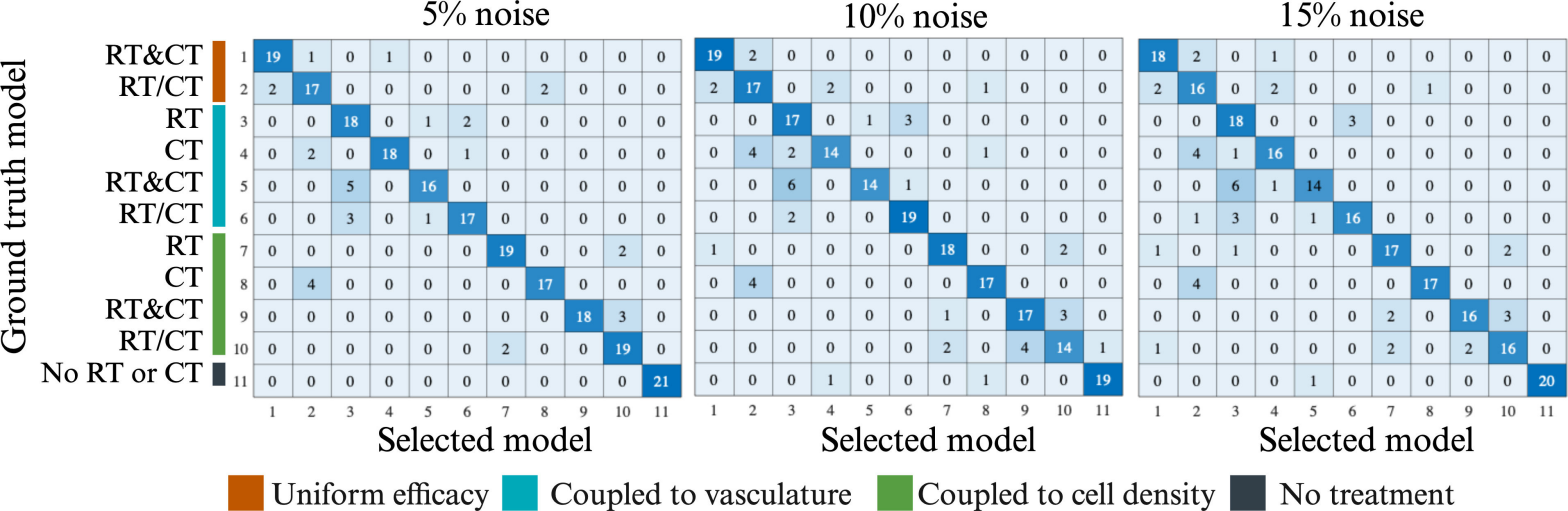
Model identifiability under noisy conditions. Each row shows the ‘ground truth’ model used to generate the synthetic tumour data for each patient, while each column tallies the model selected via the model calibration and selection framework. With 21 synthetic tumours per model, a perfectly identifiable would fill the diagonal with all 21 cases. However, in the presence of noise, we observed that each model was correctly selected for at least 14 of the 21 patients. Model 5 had the worst performance being correctly selected for 69.8% of cases. Created with BioRender.com.

The results of the model identifiability are reported in figures 3 and 4. Figure 3 shows the central slice for a representative patient, and presents the framework for generating noisy data (in this case, 5% noise added) for a given model, *M_i_*, and then fitting all 11 members of the model family to that noisy synthetic data. In this example, model *M*_3_ was used to generate the synthetic tumour and was selected as the optimal model via the AIC in equation (2.15). This process was repeated for all patients and all models, and the models selected after each run are tallied in figure 4. For 5% noise, 7 of the 11 models were correctly identified for at least 85.7% (18/21) of the cases. For the 10% noise level, only 4 of the 11 models were correctly identified at least 85.7% (18/21) of the cases. At the 15% noise level, only 3 out of 11 models were correctly identified in at least 85.7% (18/21) of cases. Across all noise levels, model 5 had the lowest performance, being correctly selected in only 69.8% (44/63) of cases, while model 11 had the highest performance, with a correct selection rate of 95.2% (60/63). Overall, our model selection framework selected the correct model in 82% (567/693) of cases. Evaluating the column sums in figure 4 for all three noise levels shows that model 3 was selected the most (a total of 82 times) while model 5 was selected the least. Generally, as noise increased the range of the column sums increased (18–26 for 5%, 15−27 for 10%, and 16−29 for 15% noise).

### (c) Accuracy of model calibrations and predictions

**Figure 5.**
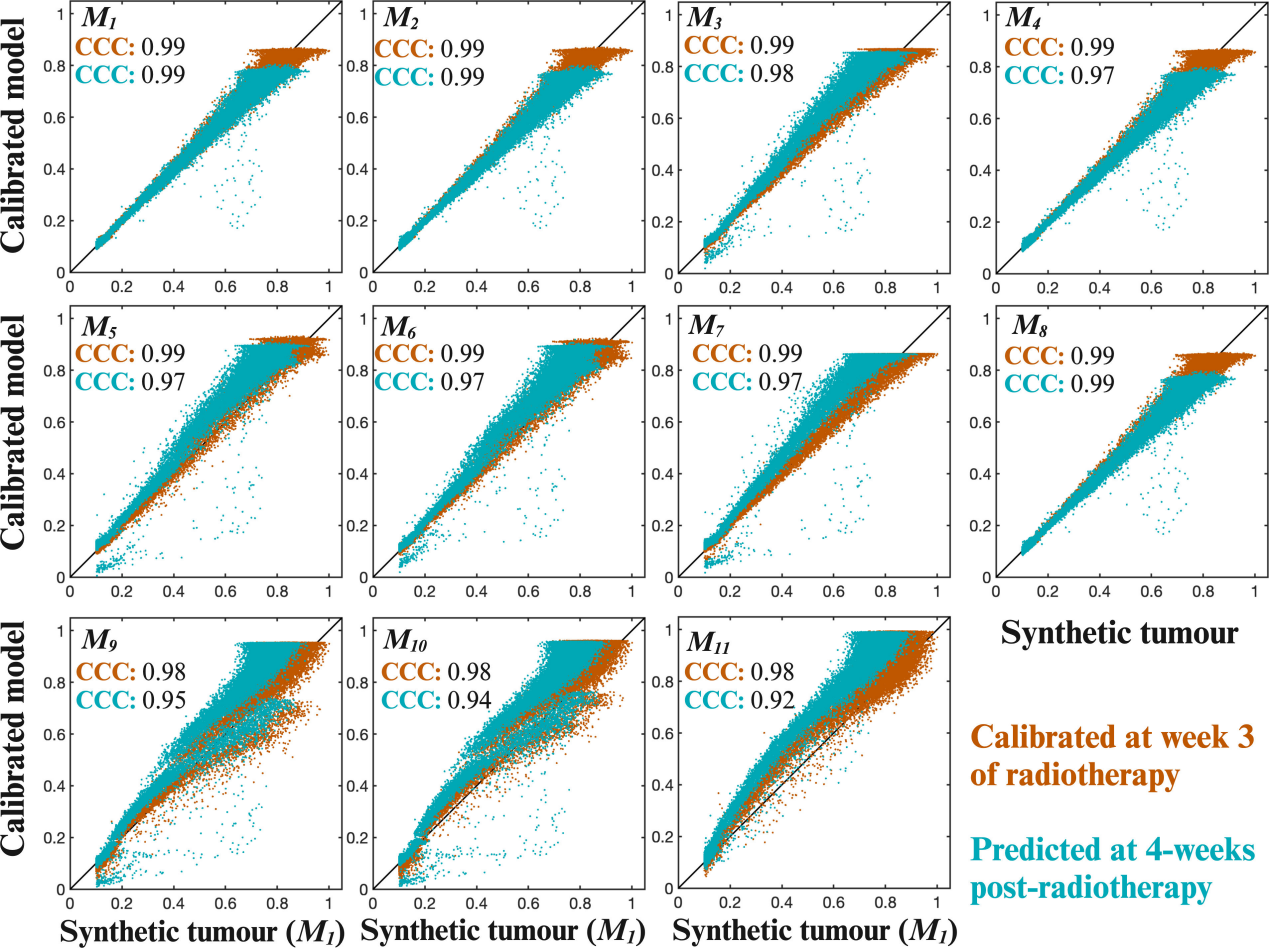
Visualization of voxel-wise accuracy of model calibrations and predictions. Results for a representative patient are shown for a synthetic tumour generated with model *M*_1_ and calibrated with all members of the model family. These results are shown for the 5% additive noise scenario. Each plot shows the calibrated model’s normalized cell density versus the synthetic tumour at the voxel level for both the last time point used for calibration (week 3 or mid-RT; in orange) and at 4 weeks post-RT (in teal) for an individual patient. All models resulted in strong agreement (CCCs > 0.98) during model calibration. When the calibrated models were used to predict future tumour growth, the agreement generally decreased (CCCs > 0.92). However, Model *M*_1_, which was used to generate the synthetic tumour, resulted in a high level of agreement for both the calibration (CCC = 0.99) and the prediction time points (CCC = 0.99). Created with BioRender.com.

Figure 5 shows and table 2 lists the error in the selected model’s estimated tumour burden at the final time point used for calibration (week 3 of RT) and at 4 weeks following RT. Figure 5 shows a visualization of the voxel-wise agreement between a single synthetic tumour (generated with model *M*_1_) and every model calibrated to that same synthetic tumour. The calibrations from each model (in orange), generally resulted in high agreement (CCCs > 0.98) with the synthetic tumour. Correlation (CCCs > 0.92) to the synthetic tumour decreased, however, for predictions made at 4 weeks post-RT. Models *M_1_, M_2_* and *M_8_* exhibited the highest agreement and correlation to the synthetic tumour with CCCs greater than 0.99 for both the calibration and prediction time points. All three of these models coupled the efficacy of RT to vasculature (table 2). The summary of results for the entire cohort is listed in table 2. For the calibration time point, the median CCC ranged from 0.70 to 0.99 with model *M*_11_ demonstrating the highest accuracy and models *M*_2_ and *M*_4_ having the lowest accuracy. The median Dice similarity values for all noise levels and models were greater than 0.97, indicating nearly identical spatial agreement between the model estimated and the ground truth synthetic tumour. Similarly, less than 1% error was observed in the model estimated TTC during calibration. For the four weeks post-RT prediction, the median CCC ranged from 0.60 to 0.98 with models *M*_2_ and *M*_*6*_ exhibiting the lowest and highest CCC values, respectively. The median Dice similarity values were greater than 0. 87 for all noise levels and models. Increased percentage error in TTC was observed for the prediction time point compared to the calibration timepoint for many of the models with a maximum error of −11.22% observed for model *M*_5_. Electronic supplementary material, table S4, lists results for comparison with table 2, showing that when the model used to generate the synthetic data was manually selected as the correct model, it yielded CCCs > 0.75, DSCs > 0.87, and percentage error in TTC < 10.25% for all noise levels and time points.

**Figure 6.**
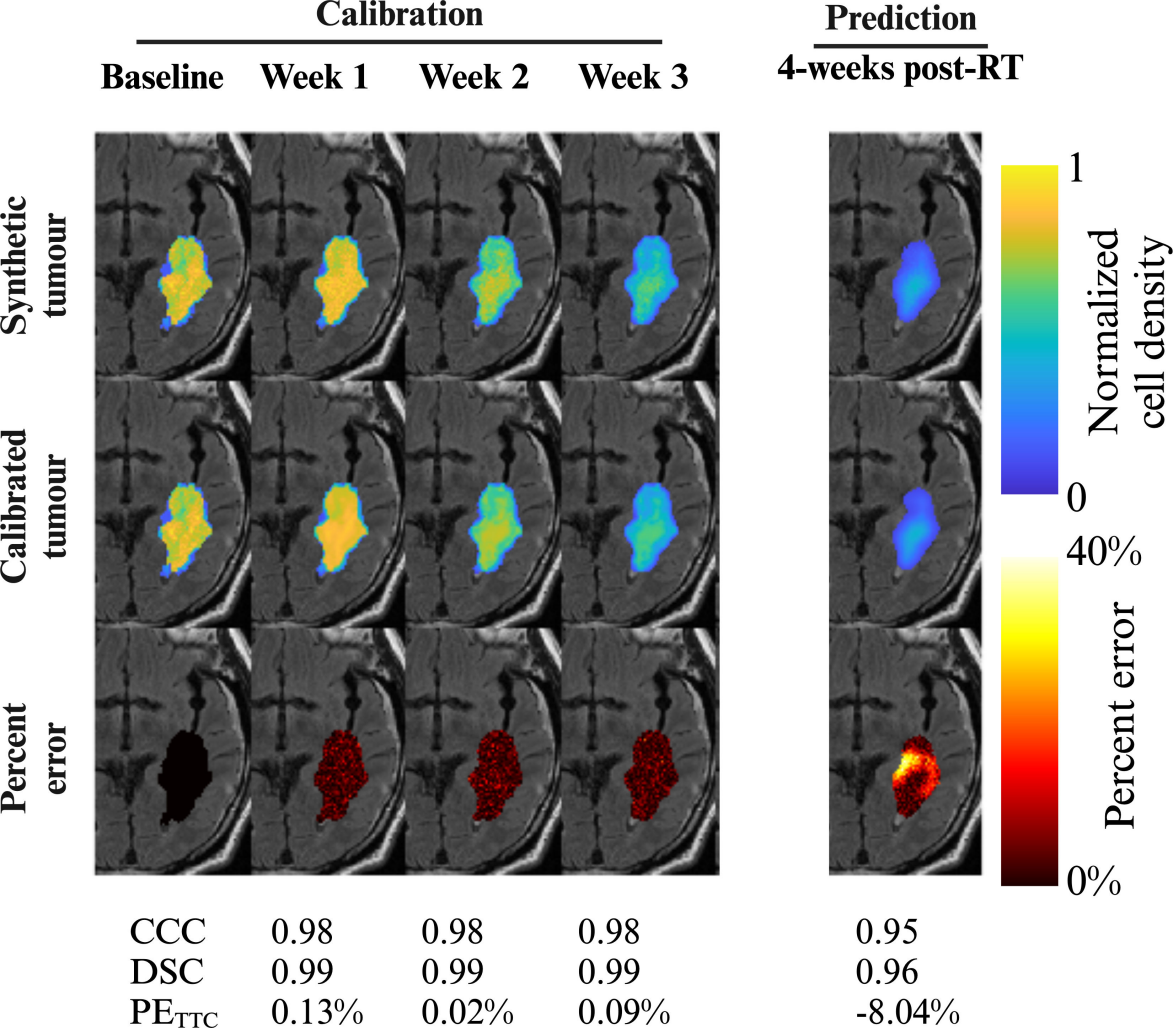
Prediction and calibration trajectory for a representative virtual patient. The top row shows the central slice of a synthetic tumour generated with model *M*_3_ with 5% noise added at all the time points used for either calibration or prediction. As expected, during treatment the tumour generally decreases in normalized cell density. The middle row shows the calibrated tumour growth trajectory for the selected model (also *M*_3_). The third row shows the percentage error at each voxel location within the tumour. Visually the trends in tumour growth and response are in agreement with the synthetic (‘ground truth’) tumour during calibration, resulting in high CCC = 0.98, DSC = 0.99, and low PE_TTC_ < 0.13%. During the prediction phase, the model underestimated the tumour cell density resulting in an increase at the voxel level (maximum percentage error of 40%, CCC = 0.95) and whole tumour level (DSC = 0.96, PE_TTC_ = −8.04%). Created with BioRender.com.

Figure 6 shows the trajectory for an individual virtual patient whose tumour was grown with model *M*_3_, and the calibration and prediction trajectories for the calibrated tumour (using the selected model *M*_3_) with 5% noise added. As the initial conditions are the same for both the synthetic and calibrated tumour, there is no error at baseline. However, the error generally increases over time, notably for the prediction at four weeks post-RT (or seven weeks past the last time point used for calibration) with a CCC of 0.95, DSC of 0.96 and a percentage error in TTC of −8.04%.

## 4. Discussion

Patient-specific mathematical models of tumour growth and response have the potential to serve as powerful tools for clinicians, enabling them to predict patient outcomes and optimize therapeutic regimens. However, without identifiable models or the ability to accurately recover model parameters, the interpretations of model predictions or optimized therapeutic regimens may be unreliable. It is difficult to know *a priori* the optimal components of a mathematical model required to characterize and predict a disease as heterogenous as cancer. Employing model selection on a family of mathematical models with varying degrees of complexity and biological assumptions offers a rigorous approach to understand what the optimal components are for an individual patient or a cohort of patients. To increase confidence in the uniqueness of the different mathematical models and the identifiability of the model parameters, we performed a systematic study on parameter and model identifiability applied to image-based models of HGG growth in a virtual cohort of 231 tumours under three noise levels. The levels of noise used for this study have additional clinical relevance in establishing confidence in model-based decisions. For example, low measurement noise may be achievable in a highly controlled clinical trial, whereas in the general clinical setting, greater noise may be expected. When 15% noise is added to the data (experimentally reasonable), we were able to accurately estimate all parameters with median error in parameters ranging from 0.04 to 72.96%. In addition, for 10 of the 11 parameters, the maximum median error calculated individually was 18.59%. Furthermore, the model used to simulate the data was identified for 82% of the cases. These results indicate that our model calibration and selection framework can distinguish the models from each other and that the parameters are uniquely identifiable. Although this study focused on HGG, the computational framework is readily adaptable to analyse model families for other disease sites [27], provided the prerequisite data aligns with that used in this study.

Assessing parameter identifiability is an important component of constructing and validating a family of mathematical models and understanding uncertainty in model parameters estimated under noisy conditions [67]. Furthermore, model parameters which are not uniquely identifiable or have large errors could be eliminated or set to a fixed or literature value thereby reducing the complexity of a model (or eliminating a model altogether). In this study, we observed that 10 of the 11 parameters were identifiable for all noise levels with less than 18.59% error. The 11th parameter, *α*/*β*, however, was poorly identifiable with errors up to 72.96%; *α*/*β* describes the sensitivity of the tumour (or tissue) to RT and its magnitude reflects the sensitivity to a fractionated RT dose. For example, larger doses per fraction are more beneficial to patients with low *α*/*β*, while smaller doses delivered over many fractions are more beneficial to patients with high *α*/*β*. Inaccurate estimation or assignment of *α*/*β* could have a substantial clinical effect if used for patient-specific optimization of RT regimens. However, we note that we and others [6,13,34,38,68] have typically assigned this to a fixed nominal value for the disease studied and recommend doing so unless the available data allows a more accurate estimation of this parameter.

Related to parameter identifiability, model identifiability seeks to determine if each member of a model family can yield unique spatial-temporal distributions of a given quantity of interest (i.e. ${\hat{N}}_{E}$ or ${\hat{N}}_{N}$ ). Model identifiability is especially important for models of treatment response which could yield drastically different interpretations of the underlying mechanism of response and influence the selection of different treatment regimens [34]. In this study, we focused our analysis on 11 models with varying assumptions on RT and CT efficacy, as a selection of the inappropriate treatment model for a given dataset could lead to different treatment decisions. We observed that our model calibration and selection framework was able to accurately identify the correct model in 82% of the cases. The most identifiable models (*M*_11_ and *M*_1_) were accurately identified in greater than 88.9% of the cases, while the least identifiable model (*M_5_*) was accurately identified for only 69.8% of the cases. Incorrect selection of the model used to generate the tumour often occurred (68.6% of the incorrect selections) within the same assumptions of treatment efficacy (e.g. *M*_3_ being selected for tumour generated with *M*_5_ which both assume treatment is coupled to vasculature). An additional cause for incorrect selection may occur when there is limited heterogeneity in the coupling term (e.g. *M*_2_ being selected for a tumour generated with *M*_8_). These values are within the range shown previously by Philips *et al*. [27] which employed a similar image-based reaction–diffusion model applied to breast cancer. When comparing the selected model to the synthetic tumour, we observed a strong agreement in the spatial overlap (DSCs ranged from 0.97 to 1.00) and weaker agreement at the voxel level (CCC ranged from 0.71 to 0.99) for calibrations. The most identifiable models, *M*_11_ and *M*_1_ *,* had DSCs greater than 0.97 and CCCs greater than 0.76. While model calibrations may have yielded accurate descriptions of tumour burden at the global and voxel levels, the calibrations did not translate to equally accurate predictions at the voxel level. Notably, the strong spatial agreement demonstrated by high DSCs may indicate that the models are less identifiable at the global level. That is, all the models provide similar predictions of the extent of the tumour burden yielding high agreement with the synthetic tumour data. This is not entirely surprising, as the underlying model for invasion and proliferation remains the same while the intra-tumour or voxel-level properties are changed between models. The effect on the intra-tumour distribution of tumour cells is observed with decreased agreement (relative to the calibration time point) at the voxel level resulting in lower CCCs and increased error in TTC. Two potential sources of this poor agreement are an inaccurate selection of a model for an individual synthetic tumour and the blurring of the model estimated $\hat{N}$ (owing to the diffusion term) relative to the synthetic tumour with non-smooth Gaussian noise added. In summary, the results at the global level suggest that the selection of the correct model is less crucial and that incorrectly selected models may still yield accurate predictions of tumour spread or location. However, if precise predictions are needed of intra-tumour cell count or TTC, it may be best to use the most identifiable models (figure 4) for the noise level expected *in vivo*. With respect to clinical applications, correct model selection would be more critical for locally directed therapies where the voxel-level accuracy is more critical versus systematic therapies where assessment and prediction of overall response will suffice to guide clinical decisions. Regarding clinical translation, we caution that this study employed *in silico* tumours whose growth and response to therapy are fully characterized by the set of partial differential equations. As a result, increased error (e.g. lower DSC or CCC) may arise in clinical settings owing to additional data variability (e.g. misregistration, imaging artefacts) and model limitations (i.e. missing critical biology phenomena).

The are several directions for future investigation for model families in mathematical oncology including sensitivity analysis, practical identifiability analysis, model weighting and uncertainty quantification. Other model analyses such as sensitivity analysis [69] or practical identifiability [24] can provide additional metrics for screening model families or guiding model composition by eliminating parameters that are insensitive (i.e. do not appreciatively influence the quantity of interest) or are not able to be precisely estimated. One systematic framework which combines sensitivity analysis, model selection, calibration and validation is OPAL [26] which methodically evaluates a model family from the fewest to most parameters divided into different Occam categories. OPAL is designed to select the simplest model (or lowest Occam category) by sequentially increasing model complexity until the desired validation criterion is met. As OPAL (and many other approaches for uncertainty quantification [17,70,71]) require Monte Carlo sampling, preliminary sensitivity analysis and parameter identifiability should be employed to reduce the number of models, parameters and overall computational costs for these approaches. One potential solution to poorly identifiable models is the use of model weighting [38,72,73] which may lessen the burden of identifying the model that best describes the data. Model weighting may use equal weighting, performance-based [72] or numerically optimized weights [74] to determine the relative contribution of each model to a forecast. In non-deterministic systems such as biology and weather forecasting, ensemble averaging or model weighting may improve the accuracy of prediction over any single model [74,75]. Alternatively, poorly identifiable models could be eliminated from the model family altogether. Finally, one important area to consider is other sources of measurement uncertainty. While we considered Gaussian noise to reflect the noise in the repeatability of these imaging measures, this study did not consider additional sources of uncertainty such as poor longitudinal registration and inconsistent or inaccurate tumour segmentations which may introduce additional uncertainty into the model framework. Future work might use Bayesian methods [17] to incorporate model and data uncertainty and provide a clearer approach for handling non-identifiable parameters. For example, non-identifiable parameters could be constrained to their prior. Indeed, Bayesian methods may be more appropriate for informing high-risk decisions—such as those in oncology. Employing Bayesian methods in future studies would also provide a clearer understanding of parameter identifiability under the constraints of the available data through analysis of posterior distributions. However, to calibrate and return predictions of highly parameterized, large-dimension models in a clinically relevant time frame, novel techniques will be required to describe parameter space (e.g. parameter maps [76]) and reduce the overall computational burden (e.g. reduced-order modelling [77]). These are areas of active research in our group and would be crucial to establishing confidence in model predictions and model-based decisions.

## 5. Conclusion

We employed a model calibration and selection framework to assess the identifiability of image-based models of HGG growth and treatment response in a virtual cohort of 231 cases. This framework was applied to the setting of adaptive RT where the goal is to deliver accurate predictions of tumour outcomes prior to the completion of therapy. Under noisy conditions we demonstrated that these models were identifiable for 82% of the tumours, were able to accurately recover 11 of the 12 parameters included in those models, and delivered accurate predictions of tumour shape (DSC > 0.95) and intra-tumour heterogeneity (CCC > 0.66). This establishes the reliability of the model calibration and selection framework as well as the expected uncertainty in the estimated parameters and model predictions.

## Data Availability

The data and code necessary to reproduce the figures and tables shown in this manuscript can be found at https://figshare.com/s/3de8fd7dec62dc4d128b. Supplementary material is available online [78].

## References

[1] Rockne RC *et al* . 2019 The 2019 mathematical oncology roadmap. Phys. Biol. **16** , 041005. (10.1088/1478-3975/ab1a09) 30991381 PMC6655440

[2] Benzekry S , Lamont C , Beheshti A , Tracz A , Ebos JML , Hlatky L , Hahnfeldt P . 2014 Classical Mathematical Models for Description and Prediction of Experimental Tumor Growth. PLoS Comput. Biol. **10** , e1003800. (10.1371/journal.pcbi.1003800) 25167199 PMC4148196

[3] Glazar DJ , Johnson M , Farinhas J , Steuer CE , Saba NF , Bonomi M , Chung CH , Enderling H . 2022 Early response dynamics predict treatment failure in patients with recurrent and/or metastatic head and neck squamous cell carcinoma treated with cetuximab and nivolumab. Oral Oncol. **127** , 105787. (10.1016/j.oraloncology.2022.105787) 35248922

[4] Swanson KR , Alvord EC Jr , Murray JD . 2000 A quantitative model for differential motility of gliomas in grey and white matter. Cell Prolif. **33** , 317–329. (10.1046/j.1365-2184.2000.00177.x) 11063134 PMC6621920

[5] Jarrett AM *et al* . 2021 Quantitative magnetic resonance imaging and tumor forecasting of breast cancer patients in the community setting. Nat. Protoc. **16** , 5309–5338. (10.1038/s41596-021-00617-y) 34552262 PMC9753909

[6] Hormuth DA II , Al Feghali KA , Elliott AM , Yankeelov TE , Chung C . 2021 Image-based personalization of computational models for predicting response of high-grade glioma to chemoradiation. Sci. Rep. **11** , 14. (10.1038/s41598-021-87887-4) 33875739 PMC8055874

[7] Alfonso JCL , Talkenberger K , Seifert M , Klink B , Hawkins-Daarud A , Swanson KR , Hatzikirou H , Deutsch A . 2017 The biology and mathematical modelling of glioma invasion: a review. J. R. Soc. Interface **14** , 20170490. (10.1098/rsif.2017.0490) 29118112 PMC5721156

[8] Mang A , Bakas S , Subramanian S , Davatzikos C , Biros G . 2020 Integrated Biophysical Modeling and Image Analysis: Application to Neuro-Oncology. Annu. Rev. Biomed. Eng. **22** , 309–341. (10.1146/annurev-bioeng-062117-121105) 32501772 PMC7520881

[9] Hormuth DA II , Farhat M , Christenson C , Curl B , Chad Quarles C , Chung C , Yankeelov TE . 2022 Opportunities for improving brain cancer treatment outcomes through imaging-based mathematical modeling of the delivery of radiotherapy and immunotherapy. Adv. Drug Deliv. Rev. **187** , 114367. (10.1016/j.addr.2022.114367) 35654212 PMC11165420

[10] Omuro A . 2013 Glioblastoma and Other Malignant Gliomas. JAMA **310** , 1842. (10.1001/jama.2013.280319) 24193082

[11] Swanson KR , Rostomily RC , Alvord EC Jr . 2008 A mathematical modelling tool for predicting survival of individual patients following resection of glioblastoma: a proof of principle. Br. J. Cancer **98** , 113–119. (10.1038/sj.bjc.6604125) 18059395 PMC2359692

[12] Scheufele K , Subramanian S , Biros G . 2021 Fully Automatic Calibration of Tumor-Growth Models Using a Single mpMRI Scan. IEEE Trans. Med. Imaging **40** , 193–204. (10.1109/tmi.2020.3024264) 32931431 PMC8565678

[13] Rockne R *et al* . 2010 Predicting the efficacy of radiotherapy in individual glioblastoma patients in vivo: a mathematical modeling approach. Phys. Med. Biol. **55** , 3271–3285. (10.1088/0031-9155/55/12/001) 20484781 PMC3786554

[14] Christenson C , Wu C , Hormuth DA II , Huang S , Bao A , Brenner A , Yankeelov TE . 2023 Predicting the spatio-temporal response of recurrent glioblastoma treated with rhenium-186 labelled nanoliposomes. Brain Multiphysics **5** , 100084. (10.1016/j.brain.2023.100084) 38187909 PMC10768931

[15] Lipkova J *et al* . 2019 Personalized Radiotherapy Design for Glioblastoma: Integrating Mathematical Tumor Models, Multimodal Scans, and Bayesian Inference. IEEE Trans. Med. Imaging **38** , 1875–1884. (10.1109/tmi.2019.2902044) 30835219 PMC7170051

[16] Corwin D , Holdsworth C , Rockne RC , Trister AD , Mrugala MM , Rockhill JK , Stewart RD , Phillips M , Swanson KR . 2013 Toward Patient-Specific, Biologically Optimized Radiation Therapy Plans for the Treatment of Glioblastoma. PLoS One **8** , e79115. (10.1371/journal.pone.0079115) 24265748 PMC3827144

[17] Chaudhuri A , Pash G , Hormuth DA II , Lorenzo G , Kapteyn M , Wu C , Lima EABF , Yankeelov TE , Willcox K . 2023 Predictive digital twin for optimizing patient-specific radiotherapy regimens under uncertainty in high-grade gliomas. Front. Artif. Intell. **6** . (10.3389/frai.2023.1222612) PMC10598726 37886348

[18] Cho H , Lewis A , Storey K , Zittle A , Cho H , Lewis A , Storey K , Zittle A . 2023 An adaptive information-theoretic experimental design procedure for high-to-low fidelity calibration of prostate cancer models. MBE **20** , 17986–18017. (10.3934/mbe.2023799) 38052545

[19] Cho H , Lewis AL , Storey KM . 2020 Bayesian Information-Theoretic Calibration of Radiotherapy Sensitivity Parameters for Informing Effective Scanning Protocols in Cancer. J. Clin. Med. **9** , 3208. (10.3390/jcm9103208) 33027933 PMC7601810

[20] Gupta S , Lee REC , Faeder JR . 2020 Parallel Tempering with Lasso for model reduction in systems biology. PLoS Comput. Biol. **16** , e1007669. (10.1371/journal.pcbi.1007669) 32150537 PMC7082068

[21] Saccomani MP , Thomaseth K . 2018 The Union between Structural and Practical Identifiability Makes Strength in Reducing Oncological Model Complexity: A Case Study. Complexity **2018** , 1–10. (10.1155/2018/2380650)

[22] Wanika L , Egan JR , Swaminathan N , Duran-Villalobos CA , Branke J , Goldrick S , Chappell M . 2024 Structural and practical identifiability analysis in bioengineering: a beginner’s guide. J. Biol. Eng. **18** , 20. (10.1186/s13036-024-00410-x) 38438947 PMC11465550

[23] Guillaume JHA *et al* . 2019 Introductory overview of identifiability analysis: A guide to evaluating whether you have the right type of data for your modeling purpose. Environ. Model. Softw. **119** , 418–432. (10.1016/j.envsoft.2019.07.007)

[24] Simpson MJ , Baker RE , Vittadello ST , Maclaren OJ . 2020 Practical parameter identifiability for spatio-temporal models of cell invasion. J. R. Soc. Interface **17** , 20200055. (10.1098/rsif.2020.0055) 32126193 PMC7115235

[25] Gerlee P . 2013 The Model Muddle: In Search of Tumor Growth Laws. Cancer Res. **73** , 2407–2411. (10.1158/0008-5472.can-12-4355) 23393201

[26] Lima EABF , Oden JT , Hormuth DA II , Yankeelov TE , Almeida RC . 2016 Selection, calibration, and validation of models of tumor growth. Math. Model. Methods Appl. Sci. **26** , 2341–2368. (10.1142/s021820251650055x) PMC5560997 28827890

[27] Phillips CM , Lima EABF , Wu C , Jarrett AM , Zhou Z , Elshafeey N , Ma J , Rauch GM , Yankeelov TE . 2023 Assessing the identifiability of model selection frameworks for the prediction of patient outcomes in the clinical breast cancer setting. J. Comput. Sci. **69** , 102006. (10.1016/j.jocs.2023.102006) PMC12330887 40777981

[28] Hormuth DA II , Jarrett AM , Feng X , Yankeelov TE . 2019 Calibrating a Predictive Model of Tumor Growth and Angiogenesis with Quantitative MRI. Ann. Biomed. Eng. **47** , 1539–1551. (10.1007/s10439-019-02262-9) 30963385 PMC6544501

[29] Slavkova KP , Patel SH , Cacini Z , Kazerouni AS , Gardner AL , Yankeelov TE , Hormuth DA II . 2023 Mathematical modelling of the dynamics of image-informed tumor habitats in a murine model of glioma. Sci. Rep. **13** , 2916. (10.1038/s41598-023-30010-6) 36804605 PMC9941120

[30] Resende ACM , Lima EABF , Almeida RC , McKenna MT , Yankeelov TE . 2022 Model selection for assessing the effects of doxorubicin on triple-negative breast cancer cell lines. J. Math. Biol. **85** , 65. (10.1007/s00285-022-01828-x) 36352309

[31] Wilson N , Drapaca CS , Enderling H , Caudell JJ , Wilkie KP . 2023 Modelling Radiation Cancer Treatment with a Death-Rate Term in Ordinary and Fractional Differential Equations. Bull. Math. Biol. **85** , 47. (10.1007/s11538-023-01139-2) 37186175 PMC10127975

[32] Mohsin N , Enderling H , Brady-Nicholls R , Zahid MU . 2023 Simulating tumor volume dynamics in response to radiotherapy: Implications of model selection. J. Theor. Biol. **576** , 111656. (10.1016/j.jtbi.2023.111656)37952611

[33] Liu Y , Suh K , Maini PK , Cohen DJ , Baker RE . 2023 Parameter identifiability and model selection for partial differential equation models of cell invasion (10.48550/arXiv.2309.01476) PMC10914513 38442862

[34] Kutuva AR , Caudell JJ , Yamoah K , Enderling H , Zahid MU . 2023 Mathematical modeling of radiotherapy: impact of model selection on estimating minimum radiation dose for tumor control. Front. Oncol. **13** , 1130966. (10.3389/fonc.2023.1130966) 37901317 PMC10600389

[35] Akaike H . 1974 A new look at the statistical model identification. Autom. Control IEEE Trans. **19** , 716–723. (10.1109/TAC.1974.1100705)

[36] Lima EABF , Oden JT , Wohlmuth B , Shahmoradi A , Hormuth DA II , Yankeelov TE , Scarabosio L , Horger T . 2017 Selection and validation of predictive models of radiation effects on tumor growth based on noninvasive imaging data. Comput. Methods Appl. Mech. Eng. **327** , 277–305. (10.1016/j.cma.2017.08.009) 29269963 PMC5734134

[37] Hormuth DA II , Weis JA , Barnes SL , Miga MI , Rericha EC , Quaranta V , Yankeelov TE . 2017 A mechanically coupled reaction–diffusion model that incorporates intra-tumoural heterogeneity to predict in vivo glioma growth. J. R. Soc. Interface **14** , 20161010. (10.1098/rsif.2016.1010) 28330985 PMC5378136

[38] Hormuth DA II , Jarrett AM , Davis T , Yankeelov TE . 2021 Towards an Image-Informed Mathematical Model of In Vivo Response to Fractionated Radiation Therapy. Cancers **13** , 1765. (10.3390/cancers13081765) 33917080 PMC8067722

[39] Yan D . 2010 Adaptive Radiotherapy: Merging Principle Into Clinical Practice. Semin. Radiat. Oncol. **20** , 79–83. (10.1016/j.semradonc.2009.11.001) 20219545

[40] Sonke JJ , Aznar M , Rasch C . 2019 Adaptive Radiotherapy for Anatomical Changes. Semin. Radiat. Oncol. **29** , 245–257. (10.1016/j.semradonc.2019.02.007) 31027642

[41] Enderling H , Alfonso JCL , Moros E , Caudell JJ , Harrison LB . 2019 Integrating Mathematical Modeling into the Roadmap for Personalized Adaptive Radiation Therapy. Trends Cancer **5** , 467–474. (10.1016/j.trecan.2019.06.006) 31421904

[42] Jarrett AM , Faghihi D , Hormuth DA II , Lima EABF , Virostko J , Biros G , Patt D , Yankeelov TE . 2020 Optimal Control Theory for Personalized Therapeutic Regimens in Oncology: Background, History, Challenges, and Opportunities. J. Clin. Med. **9** , 1314. (10.3390/jcm9051314) 32370195 PMC7290915

[43] Baldock AL *et al* . 2013 From Patient-Specific Mathematical Neuro-Oncology to Precision Medicine. Front. Oncol. **3** , 62. (10.3389/fonc.2013.00062) 23565501 PMC3613895

[44] Hogea C , Davatzikos C , Biros G . 2008 An image-driven parameter estimation problem for a reaction–diffusion glioma growth model with mass effects. J. Math. Biol. **56** , 793–825. (10.1007/s00285-007-0139-x) 18026731 PMC2871396

[45] Lipková J , Menze B , Wiestler B , Koumoutsakos P , Lowengrub JS . 2022 Modelling glioma progression, mass effect and intracranial pressure in patient anatomy. J. R. Soc. Interface **19** , 20210922. (10.1098/rsif.2021.0922) 35317645 PMC8941421

[46] Konukoglu E , Clatz O , Menze B , Stieltjes B , Weber MA , Mandonnet E , Delingette H , Ayache N . 2010 Image Guided Personalization of Reaction-Diffusion Type Tumor Growth Models Using Modified Anisotropic Eikonal Equations. Med. Imaging IEEE Trans. **29** , 77–95. (10.1109/TMI.2009.2026413) 19605320

[47] El-Hachem M , McCue SW , Simpson MJ . 2020 A sharp-front moving boundary model for malignant invasion. Phys. D **412** , 132639. (10.1016/j.physd.2020.132639)

[48] Green MA , Bilston LE , Sinkus R . 2008 In vivo brain viscoelastic properties measured by magnetic resonance elastography. NMR Biomed. **21** , 755–764. (10.1002/nbm.1254) 18457350

[49] Mabray MC , Barajas RF Jr , Cha S . 2015 Modern Brain Tumor Imaging. Brain Tumor Res. Treat. **3** , 8. (10.14791/btrt.2015.3.1.8) 25977902 PMC4426283

[50] Garg I , Miga MI . 2008 Preliminary investigation of the inhibitory effects of mechanical stress in tumor growth. In Proc. SPIE, San Diego, CA, pp. 69182L–69182L. (10.1117/12.773376)

[51] Narasimhan S , Johnson HB , Nickles TM , Miga MI , Rana N , Attia A , Weis JA . 2019 Biophysical model‐based parameters to classify tumor recurrence from radiation‐induced necrosis for brain metastases. Med. Phys. **46** , 0. (10.1002/mp.13461) PMC6510636 30816555

[52] Jbabdi S , Mandonnet E , Duffau H , Capelle L , Swanson KR , Pélégrini‐Issac M , Guillevin R , Benali H . 2005 Simulation of anisotropic growth of low‐grade gliomas using diffusion tensor imaging. Magn. Reson. Med. **54** , 616–624. (10.1002/mrm.20625) 16088879

[53] Painter KJ , Hillen T . 2013 Mathematical modelling of glioma growth: The use of Diffusion Tensor Imaging (DTI) data to predict the anisotropic pathways of cancer invasion. J. Theor. Biol. **323** , 25–39. (10.1016/j.jtbi.2013.01.014) 23376578

[54] Hormuth DA 2nd , Eldridge SL , Weis JA , Miga MI , Yankeelov TE . 2018 Mechanically Coupled Reaction Diffusion Model to Predict Glioma Growth: Methodological Details. In Springer methods and protocols: cancer systems biology (ed. L von Stechow ), pp. 225–241, vol. **1711** . New York, NY: Springer New York. (10.1007/978-1-4939-7493-1_11) PMC6530463 29344892

[55] Hormuth DA II , Weis JA , Barnes SL , Miga MI , Quaranta V , Yankeelov TE . 2018 Biophysical Modeling of In Vivo Glioma Response After Whole-Brain Radiation Therapy in a Murine Model of Brain Cancer. Int. J. Radiat. Oncol. **100** , 1270–1279. (10.1016/j.ijrobp.2017.12.004) PMC5934308 29398129

[56] Hormuth DA II , Jarrett AM , Yankeelov TE . 2020 Forecasting tumor and vasculature response dynamics to radiation therapy via image based mathematical modeling. Radiat. Oncol. **15** , 4. (10.1186/s13014-019-1446-2) 31898514 PMC6941255

[57] Prokopiou S *et al* . 2015 A proliferation saturation index to predict radiation response and personalize radiotherapy fractionation. Radiat. Oncol. **10** , 8. (10.1186/s13014-015-0465-x) 26227259 PMC4521490

[58] Stupp R *et al* . 2005 Radiotherapy plus Concomitant and Adjuvant Temozolomide for Glioblastoma. N. Engl. J. Med. **352** , 987–996. (10.1056/nejmoa043330) 15758009

[59] Chang EL *et al* . 2007 Evaluation of Peritumoral Edema in the Delineation of Radiotherapy Clinical Target Volumes for Glioblastoma. Int. J. Radiat. Oncol. **68** , 144–150. (10.1016/j.ijrobp.2006.12.009) 17306935

[60] Hagmann P , Jonasson L , Maeder P , Thiran JP , Wedeen VJ , Meuli R . 2006 Understanding Diffusion MR Imaging Techniques: From Scalar Diffusion-weighted Imaging to Diffusion Tensor Imaging and Beyond. RadioGraphics **26** , S205–S223. (10.1148/rg.26si065510) 17050517

[61] Liu T , Li H , Wong K , Tarokh A , Guo L , Wong STC . 2007 Brain tissue segmentation based on DTI data. NeuroImage **38** , 114–123. (10.1016/j.neuroimage.2007.07.002) 17804258 PMC2430665

[62] Hoopes A , Mora JS , Dalca AV , Fischl B , Hoffmann M . 2022 SynthStrip: skull-stripping for any brain image. NeuroImage **260** , 119474. (10.1016/j.neuroimage.2022.119474) 35842095 PMC9465771

[63] Phan T , Bennett J , Patten T . 2023 Practical Understanding of Cancer Model Identifiability in Clinical Applications. Life **13** , 410. (10.3390/life13020410) 36836767 PMC9961656

[64] Levenberg K . 1944 A method for the solution of certain non-linear problems in least squares. Q. J. Appl. Math. **II** , 164–168. (10.1090/qam/10666)

[65] Martin I , Dozin B , Quarto R , Cancedda R , Beltrame F . 1997 Computer-based technique for cell aggregation analysis and cell aggregation in in vitro chondrogenesis. Cytometry **28** , 141–146. (10.1002/(sici)1097-0320(19970601)28:23.0.co;2-i) 9181304

[66] Lin LIK . 1989 A Concordance Correlation Coefficient to Evaluate Reproducibility. Biometrics **45** , 255. (10.2307/2532051) 2720055

[67] Chis OT , Banga JR , Balsa-Canto E . 2011 Structural Identifiability of Systems Biology Models: A Critical Comparison of Methods. PLoS One **6** , e27755. (10.1371/journal.pone.0027755) 22132135 PMC3222653

[68] Rockne RC *et al* . 2015 A patient-specific computational model of hypoxia-modulated radiation resistance in glioblastoma using 18F-FMISO-PET. J. R. Soc. Interface **12** , 20141174. (10.1098/rsif.2014.1174) 25540239 PMC4305419

[69] Lorenzo G , Jarrett AM , Meyer CT , DiCarlo JC , Virostko J , Quaranta V , Tyson DR , Yankeelov TE . 2023 A global sensitivity analysis of a mechanistic model of neoadjuvant chemotherapy for triple negative breast cancer constrained by in vitro and in vivo imaging data. Eng. Comput. (10.1007/s00366-023-01873-0) PMC11607094 39620056

[70] Zahid MU , Mohsin N , Mohamed ASR , Caudell JJ , Harrison LB , Fuller CD , Moros EG , Enderling H . 2021 Forecasting Individual Patient Response to Radiation Therapy in Head and Neck Cancer With a Dynamic Carrying Capacity Model. Int. J. Radiat. Oncol. **111** , 693–704. (10.1016/j.ijrobp.2021.05.132) PMC8463501 34102299

[71] Hawkins-Daarud A , Johnston SK , Swanson KR . 2019 Quantifying Uncertainty and Robustness in a Biomathematical Model–Based Patient-Specific Response Metric for Glioblastoma. JCO Clin. Cancer Informatics 1–8. (10.1200/cci.18.00066) PMC6633916 30758984

[72] Wagenmakers EJ , Farrell S . 2004 AIC model selection using Akaike weights. Psychon. Bull. Rev. **11** , 192–196. (10.3758/bf03206482) 15117008

[73] Uster DW , Stocker SL , Carland JE , Brett J , Marriott DJE , Day RO , Wicha SG . 2021 A Model Averaging/Selection Approach Improves the Predictive Performance of Model‐Informed Precision Dosing: Vancomycin as a Case Study. Clin. Pharmacol. Ther. **109** , 175–183. (10.1002/cpt.2065) 32996120

[74] Peña M , van den Dool H . 2008 Consolidation of Multimodel Forecasts by Ridge Regression: Application to Pacific Sea Surface Temperature. J. Clim. **21** , 6521–6538. (10.1175/2008jcli2226.1)

[75] Kharin VV , Zwiers FW . 2003 Improved Seasonal Probability Forecasts. J. Clim. **16** , 1684–1701. (10.1175/1520-0442(2003)0162.0.co;2)

[76] Liang B , Tan J , Lozenski L , Hormuth DA , Yankeelov TE , Villa U , Faghihi D . 2023 Bayesian Inference of Tissue Heterogeneity for Individualized Prediction of Glioma Growth. IEEE Trans. Med. Imaging , 1–1. **42** , 2865–2875. (10.1109/tmi.2023.3267349) PMC10599765 37058375

[77] Christenson C , Wu C , Hormuth DA II , Stowers CE , LaMonica M , Ma J , Rauch GM , Yankeelov TE . 2024 Fast model calibration for predicting the response of breast cancer to chemotherapy using proper orthogonal decomposition. J. Comput. Sci. **82** , 102400. (10.1016/j.jocs.2024.102400) 40303598 PMC12037169

[78] Hiremath KC , Atakishi K , Bueno da Fonseca Lima EA , Farhat M , Panthi B , Langshaw H *et al* . 2025 Supplementary material from: Identifiability and model selection frameworks for models of high-grade glioma response to chemoradiation. Figshare. (10.6084/m9.figshare.c.7652257) PMC12105797 40172557

